# Phycobilins as Potent Food Bioactive Broad-Spectrum Inhibitors Against Proteases of SARS-CoV-2 and Other Coronaviruses: A Preliminary Study

**DOI:** 10.3389/fmicb.2021.645713

**Published:** 2021-06-10

**Authors:** Brahmaiah Pendyala, Ankit Patras, Chandravanu Dash

**Affiliations:** ^1^Department of Agricultural and Environmental Sciences, Food Science Program, College of Agriculture, Tennessee State University, Nashville, TN, United States; ^2^Meharry Medical College, Nashville, TN, United States

**Keywords:** food bioactive constituents, broad-spectrum inhibitors, coronaviruses, SARS-CoV-2, COVID-19, main protease, papain-like protease

## Abstract

In the 21st century, we have witnessed three coronavirus outbreaks: SARS in 2003, MERS in 2012, and the ongoing pandemic coronavirus disease 2019 (COVID-19). The search for efficient vaccines and development and repurposing of therapeutic drugs are the major approaches in the COVID-19 pandemic research area. There are concerns about the evolution of mutant strains (e.g., VUI – 202012/01, a mutant coronavirus in the United Kingdom), which can potentially reduce the impact of the current vaccine and therapeutic drug development trials. One promising approach to counter the mutant strains is the “development of effective broad-spectrum antiviral drugs” against coronaviruses. This study scientifically investigates potent food bioactive broad-spectrum antiviral compounds by targeting main protease (M^pro^) and papain-like protease (PL^pro^) proteases of coronaviruses (CoVs) using *in silico* and *in vitro* approaches. The results reveal that phycocyanobilin (PCB) shows potential inhibitor activity against both proteases. PCB had the best binding affinity to Mpro and PLpro with IC_50_ values of 71 and 62 μm, respectively. Also, *in silico* studies with M^pro^ and PL^pro^ enzymes of other human and animal CoVs indicate broad-spectrum inhibitor activity of the PCB. As with PCB, other phycobilins, such as phycourobilin (PUB), phycoerythrobilin (PEB), and phycoviolobilin (PVB) show similar binding affinity to SARS-CoV-2 M^pro^ and PL^pro^.

## Introduction

Coronaviruses (CoVs) belongs to the subfamily of Orthocoronavirinae, family Coronavidae, order Nidovirales. They are large (average diameter of 120 nm), enveloped, positive-sense single-stranded RNA viruses with a genome size of ∼26 to 32 kb (Woo et al., 2010). Based on antigen cross-reactivity and genetic makeup, four subgroups (alpha, beta, gamma, and delta) are subdivided into 26 different species of CoVs (Cleri et al., 2010). CoVs cause diseases in mammals and birds; alpha and beta group CoVs are pathogenic to humans (Paules et al., 2020). The seven CoVs that can cause infectious diseases in humans are HCoV-229E, HCoV-NL63, HCoV-OC43, HCoV-HKU1, severe acute respiratory syndrome coronavirus (SARS-CoV), Middle East respiratory virus coronavirus (MERS-CoV), and 2019-nCoV (2019-novel coronavirus) or SARS-CoV-2 (Hamre and Procknow, 1966; McIntosh et al., 1967; Drosten et al., 2003; van der Hoek et al., 2004; Woo et al., 2005; Bermingham et al., 2012; Wu F. et al., 2020). The first four common CoVs persistently circulate in humans and are responsible for 10–30% of common colds (Paules et al., 2020). The other three deadly viruses are etiological agents of fatal respiratory syndromes SARS, MERS, and coronavirus disease 2019 (COVID-19), respectively. The SARS epidemic in 2003 ended with 8098 reported cases, 774 deaths (fatality rate 9.7%), whereas the MERS outbreak in 2012 caused 2494 reported cases, 858 deaths (fatality rate 34%) (World Health Organization (WHO), 2003; Alfaraj et al., 2019). COVID-19, the current pandemic outbreak, first identified in 2019, report >37.1 million confirmed cases with >1.07 million deaths (fatality rate 2.9%) as of October 12, 2020 (World Health Organization (WHO), 2020). Avian infectious bronchitis virus (IBV), feline infectious peritonitis virus (FIPV), canine CoV, and porcine transmissible gastroenteritis virus (TGEV) cause respiratory and enteric diseases in farm and domestic pet animals (Pratelli, 2006; Cavanagh, 2007; Pedersen, 2009; Odend’hal, 2012).

Till now, there are no approved vaccines and therapeutic drugs for COVID-19 or other human coronavirus infections and a lack of enough clinical trial data to make treatment decisions. Although vaccines have been developed against animal viruses IBV, canine CoV, and TGEV to help prevent serious diseases (Park et al., 1998; Carmichael, 1999; Liu and Kong, 2004), there are some potential problems, such as recombination events between field and vaccine strains, the emergence of novel serotypes, and antibody-dependent enhancement remain. The rapid development of vaccines and repurposing of approved antivirals drugs (e.g., remdesivir) are major clinical approaches of a pandemic preparedness plan. The development of broad-spectrum antiviral agents that are effective against a wide range of CoVs and other classes of viruses, including emerging ones, could be a promising strategy (Bekerman and Einav, 2015; Fauci and Morens, 2016; Cho and Glenn, 2020).

Broad-spectrum antiviral targeting strategies can be classified into two categories: (i) entry inhibitors that interact with existing virus particles outside of cells and prevent infection (Hangartner et al., 2006) and (ii) replication inhibitors aimed at stopping viral genome replication to curtail production of new virus particles (De Clercq, 2004). The S glycoprotein of coronaviruses, the main determinant of host cell attachment and viral entry, is not well conserved between HCoVs (Totura and Bavari, 2019). On the other hand, CoV non-structural proteins (nsps) are highly conserved components of the coronavirus life cycle that mediate viral replication (Totura and Bavari, 2019). Literature studies report the following SARS-CoV-2 nsp targets; main protease (M^pro^), papain-like protease (PL^pro^), Nsp3, RdRp, Helicase, Nsp14, Nsp15, Nsp16, N protein to inhibit virus replication (Wu C. et al., 2020). Proteolytic processing of viral polyproteins into functional nsps by two viral proteases, the M^pro^ and PL^pro^, is an important event of the CoV life cycle. The M^pro^ acts on minimum 11 cleavage sites of replicase 1ab, ∼790 kDa; at recognition sequence Leu-Gln↓ (Ser, Ala, Gly) (↓ indicates cleavage site), most cleavage sites block viral replication (Zhang et al., 2020). PL^Pro^ enzyme hydrolyses the peptide bond at the carboxyl side of glycine (P1 position) and releases nsp1, nsp2, and nsp3 functional proteins, which play a key role in viral replication (Rut et al., 2020). Therefore, these proteases would be potential targets for the development of broad-spectrum antiviral drugs. CoVs M^pro^ and PL^pro^ crystal structures are available for public access in the protein data bank (PDB).

Natural food bioactive compounds are gaining importance as supplementary antiviral therapeutic compounds in the modern healthcare sector because of their lower toxicity and fewer side effects, additional health benefits (antioxidant, anti-inflammatory, and immunomodulation activities), and potential use in conjunction with preexisting therapies. Several literature studies report antiviral properties of food bioactive compounds against CoVs and other viruses (Table 1; Ghildiyal et al., 2020; Mani et al., 2020). In view of the issues posed above, identifying natural food bioactive broad-spectrum antiviral agents against the CoVs is a more reasonable and attractive prospect and could provide an effective first line of defense against future emerging CoVs related diseases. Herein, we report the phycobilins as potent food bioactive broad-spectrum inhibitor compounds against M^pro^ and PL^pro^ of SARS-CoV-2 and other CoVs via *in silico* and *in vitro* approaches.

**TABLE 1:** Antiviral properties of selected food bioactive constituents.

Bioactive compound	Antiviral activity	References
Phycocyanobilin	Spirulina extract exhibited anti-flu efficacy against wide range of influenza viruses with EC_50_ values from 0.58 to 1.17 mg/mL.	Chen et al., 2016
Quercetin	Inhibited hepatitis C virus production almost completely (>95%) at concentration of 10 μM.	Bachmetov et al., 2012
Riboflavin	In combination with poly r(A-U) showed 7 to 12-fold antiviral activity against human foreskin fibroblast-vesicular stomatitis virus	Jamison et al., 1990
Cyanidin	Cyanidin-3-sambubiocide was found to be a potent inhibitor for H1N1 neuraminidase (NA) activity with IC_50_ value 72 μM.	Kannan and Kolandaivel, 2018
Daidzein	Exhibited anti-dengue activity with IC_50_ = 142.6 μg mL^–1^ against DENV-2.	Zandi et al., 2011
Genistein	Reduced hepatitis B virus production with an IC_50_ value of 33 and 46 μM for human and macaque fibroblasts, respectively.	LeCher et al., 2019
Catechin	Catechins (−)-epigallocatechin gallate (EGCG), (−)-epicatechin gallate (ECG) were identified as potent inhibitors of influenza virus replication with EC_50_ of 22–28 and 22–40 μM, respectively.	Song et al., 2005
Resveratrol	MERS-CoV titer reduced 4 logs by resveratrol treatment at 250 μM concentration after 48 h of infection.	Lin et al., 2017
Curcumin	Curcumin and its derivatives showed antiviral effects on HSV-1 in cell culture with IC_50_ values in range of 13.9–33.0 μg/mL.	Zandi et al., 2010
Astaxanthin	Pre-treatment of Vero cells with 75 μg mL^–1^ of *Haematococcus pluvialis* ethanol extract with carotenoids inhibited Herpes simplex virus type 1 (HSV-1) infection by approximately 85%.	Santoyo et al., 2012
β-carotene	Pre-treatment of Vero cells with 75 μg mL-1 of *H. pluvialis* ethanol extract with carotenoids inhibited Herpes simplex virus type 1 (HSV-1) infection by approximately 85%.	Santoyo et al., 2012
Capsaicin	Methanolic extract of *Capsicum annuum* exhibited a considerable anti-HSV-1 and anti-HSV-2 activities at the concentration of 25 μg/mL.	Hafiz et al., 2017
Gingerol	Fresh *Zingiber officinale* inhibited human respiratory syncytial virus (HRSV) with IC_50_ of 144.9 μg/mL in HEp-2 cells and 73.3 μg/ml in A549 cells.	San Chang et al., 2013
Vanillin	MY21 (a vanillin derivative) had the IC_50_ of 50 μM against H1N1 neuraminidase (NA).	Hariono et al., 2016
Eugenol	IC_50_ values for the anti-HSV effects of eugenol were 25.6 and 16.2 μg/mL for HSV-1 and HSV-2, respectively.	Benencia and Courreges, 2000
Thymol	Exhibited significant antiviral activity with an IC*50* of 7 μM against herpes simplex virus type I.	Lai et al., 2012

## Materials and Methods

### *In silico* Screening of Inhibitor Compounds

#### Preparation of Protein and Ligand for Docking

The crystal structures of M^pro^ (PDB ID – 6LU7) and PL^pro^ of SARS-CoV-2 (PDB ID – 6WUU) and other CoVs used in this study were obtained from the RCSB PDB. Ligand structures were obtained from Pubchem and Chemical Entities of Biological Interest (ChEBI) as SDF format, Open Babel was used for format transformation or 3-D coordinate generation for the uploaded files (O’Boyle et al., 2011). The MGLTools were used to delete other chains, and heteroatoms (included water), adding missing atoms, hydrogens, and charges. Further, the pdbqt files were prepared for proteins and ligands binding.

#### Molecular Docking and Molecular Simulation Studies

Autodock Vina was used as a docking engine. It is critical to define the docking grid box appropriately due to the small molecule docking procedure (Trott and Olson, 2010). The docking box is defined as the center of native ligand coordinates with 40 Å × 40 Å × 40 Å in length to include the residues of the entire cavity, and the exhaustiveness level was set on 12 with number of modes 10. For visualization, the docking results PDBQT files were exported, and docked protein-ligand complex structures were visualized using Pymol. Active site residues within 3 or 3.5 Å of ligand and polar contacts were determined with this same tool. The ligand docking procedure was validated by redocking of the native ligand with the same protocol and the grid parameters as used for food bioactive compounds. The redocked ligand was then superimposed onto the reference co-crystallized ligand complex using Pymol, and the root mean square deviation (RMSD) was analyzed.

Molecular dynamic simulations were performed using NAMD (Phillips et al., 2005). The parameters, structure, and topology files for the ligand were generated using the CHARMM-GUI Web server (Jo et al., 2008). Visual molecular dynamics (VMD) was used to generate protein structure (PSF) files (Humphrey et al., 1996). Each protein-ligand docked complex was solvated and ionized with 0.15 M ions (Na+ and Cl−) to neutralize the charge and electrostatic screening. The systems were subjected to 2000 steps of steepest descent energy minimization before a production run at the NPT of 0.5 ns (250,000 steps). The temperature (310 K) and pressure (1 atm) were controlled by the Langevin and Langevin piston methods (Feller et al., 1995). VMD software was used to visualize simulations and to analyze average ligand-RMSD and protein-RMSD and hydrogen bonds.

### *In vitro* Enzymatic Assays

For enzyme inhibition studies, selected phytochemicals, PCB, Quercetin, Riboflavin, Cyanidin, Daidzein, and Genistein, were purchased from Santa Cruz Biotechnology (Santa Cruz, CA, United States). Enzyme assay kits, 3CL Protease, MBP-tagged (SARS-CoV-2) assay (Catalog #79955), and papain-like protease (SARS-CoV-2) assay kit: protease activity (Catalog #79995), were purchased from BPS Bioscience (San Diego, CA, United States).

#### M^pro^ Assay

Fluorescence resonance energy transfer (FRET)-based cleavage assay (Zhu et al., 2011) was used for *in vitro* enzyme inhibition study. SARS-CoV-2 M^pro^ or 3CL Protease, GenBank Accession No. YP_009725301, amino acids 1-306 (full length), with an N-terminal MBP-tag, expressed in an *Escherichia coli* and its fluorescent substrate with cleavage site (indicated by ↓) DABCYL-KTSAVLQ↓SGFRKME-EDANS, inhibitor control (GC376), and the assay buffer composed of 20 mM Tris, 100 mM NaCl, 1 mM EDTA, 1 mM DTT, pH 7.3 were used. Initially, 15 μL of the SARS-CoV-2 M^pro^ in reaction buffer at the final concentration of 10 ng/μL and 5 μL of inhibitor control (GC376, final concentration 50 μM)/test inhibitor (10–600 μM)/inhibitor solvent (positive control) was pipetted into a 384-well plate. Stock solutions of the compounds were prepared with 100% DMSO. Afterward, the plate was preincubated for 30 min at room temperature with slow shaking. The enzymatic reaction was then initiated by adding of 5 μL of the substrate dissolved in the reaction buffer to 25 μL final volume (final concentration 50 μM) and incubated at room temperature for 4 h. The fluorescence signal of the Edans generated due to the cleavage of the substrate by the M^pro^ was monitored at excitation at 360 nm with an emission wavelength of 460 nm, using a spectrophotometric microplate reader (Synergy H1 Hybrid Multi-Mode Reader; BioTek Instruments, Inc., Winooski, VT, United States).

#### PL^pro^ Assay

Severe acute respiratory syndrome coronavirus-2 PL^pro^ (papain-like protease), GenBank Accession No. QHD43415, amino acids 1564–1882, with N-terminal His-tag, expressed in an *E. coli* and its fluorescent substrate Z-Arg-Leu-Arg-Gly-Gly-AMC, inhibitor control (GRL0617) and the assay buffer (40 mM Tris pH 8, 110 mM NaCl, 1 mM DTT) was used for the inhibition assay. Briefly, 30 μL of the SARS-CoV-2 PL^pro^ in reaction buffer at the final concentration of 0.44 ng/μL and 10 μL of inhibitor control (GRL0617, final concentration 100 μM)/test inhibitor (10–600 μM)/inhibitor solvent (positive control) was pipetted into a 96-well plate. Afterward, the plate was preincubated for 30 min at room temperature with slow shaking. The enzymatic reaction was then initiated by the addition of 10 μL of the substrate dissolved in the reaction buffer to 50 μL final volume (final concentration 25 μM), incubated at room temperature for 40–60 min. The fluorescence signal of the substrate after the enzymatic reaction was monitored at an excitation at 360 nm with an emission wavelength of 460 nm, using a spectrophotometric microplate reader (Synergy H1 Hybrid Multi-Mode Reader; BioTek Instruments, Inc., Winooski, VT, United States). Triplicate experiments (*N* = 3) were performed for both M^pro^ and PL^pro^ assays, and the mean value was presented with ± standard deviation (SD).

## Results

### Selection of Phytochemicals for the Study

A total of 16 phytochemicals from different chemical classes were selected based on the previous reports of their potent antiviral effects (Table 1): linear tetrapyrrole – phycocyanobilin (PCB), flavonols – quercetin, catechin, flavin – riboflavin, anthocyanin – cyanidin, isoflavones – daidzein, genistein, stilbenoid phenol – resveratrol, linear diarylheptanoid – curcumin, Xanthophyll – astaxanthin, carotenes – β-carotene, phenolic alkaloid – capsaicin, phenolic ketone – gingerol, phenolic aldehyde – vanillin, allylbenzene – eugenol, monoterpenoid phenol – thymol.

### *In silico* Binding Interaction Studies of Selected Phytochemical Compounds With SARS-CoV-2 M^pro^ and PL^pro^

**FIGURE 1:**
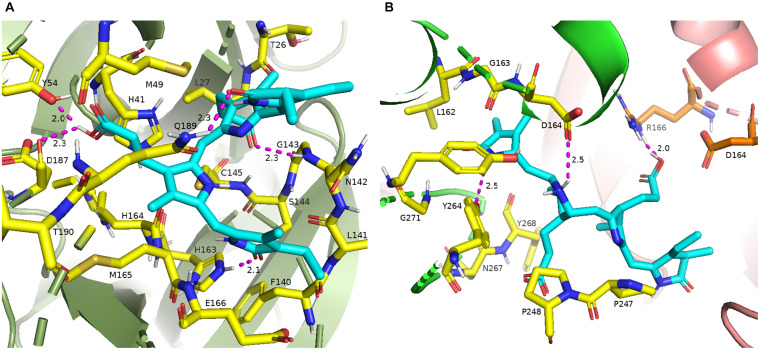
**(A)** 3-D binding pocket of SARS-CoV-2 M^pro^ with top model PCB (cyan color), surrounding active site amino acid residues (yellow color) within 3 Å; remaining residues are represented as a cartoon; **(B)** 3-D binding pocket of SARS-CoV-2 PL^pro^ with top model PCB (cyan color), surrounding active site amino acid residues (chain A, yellow color; chain B, orange color) within 3 Å; remaining residues are represented as a cartoon (chain A, green color; chain B, light pink color). Polar interactions are represented as magenta color.

The 16 selected phytochemicals were docked into the active site pocket of SARS-CoV-2 M^pro^ and PL^pro^. Table 2 depicts the source, docking score, and polar contacts of selected phytochemical bioactive compounds with binding site amino acid residues of SARS-CoV-2 proteases. For M^pro^, the results show PCB docked with the best score or binding energy of −8.6 Kcal/mol followed by Riboflavin (−7.9 Kcal/mol), Cyanidin (−7.9 Kcal/mol), Daidzein (−7.8 Kcal/mol), and Genistein (−7.6 Kcal/mol). Twelve key active-site amino acid residues (Tyr 54, Gly 143, His 163, Asp 187, Gln 189, Glu 166, Cys 145, Leu 141, Ser 144, Thr 26, Gln 192, and Thr 190) of SARS-CoV-2 M^pro^ involved in polar interactions at a distance of ≤3 Å with ligand phytochemical compounds. Specific polar contacts of each phytochemical compound are shown in Table 2. In the case of PL^pro^, as the reported peptide inhibitor VIR250 is bound to the dimer interface in the crystal structure of 6WUU (Rut et al., 2020), the docking studies were performed with dimer form. Similarly, PCB docked with the best score or binding energy of −9.8 Kcal/mol followed by Astaxanthin, (−9.3 Kcal/mol), β-carotene (−9.2 Kcal/mol), Daidzein (−8.9 Kcal/mol), Riboflavin (−8.5 Kcal/mol), and Genistein (−8.3 Kcal/mol). Eleven key active site amino acid residues (Asp 164, Tyr 264, Gln 269, Arg 166, Tyr 273, Glu 161, Tyr 268, Lys 157, Leu 162, Gly 266, and Ser 170) in chain A and 13 amino acid residues (Arg 166, Gln 174, Met 208, Glu 161, Glu 167, Cys 155, Lys 232, Met 206, Arg 183, Glu 203, Tyr 268, Tyr 273, and Thr 301) in chain B of SARS-CoV-2 PL^pro^ are involved in polar interactions at a distance of ≤3 Å with ligand phytochemical compounds. Table 2 illustrates the specific polar contacts between phytochemical compounds and proteases. Figure 1 shows a 3-D representation of the binding pocket of M^pro^ and PL^pro^ with top score model pose of PCB. The co-crystalized structure of native 6LU7-N3 and 6WUU-VIR250 complexes and polar contacts are represented in Supplementary Figures 1a, 2a. The docking validation studies reveal that both N3 peptide inhibitor and VIR250 bound exactly to the active site of 6LU7 and 6WUU, respectively. Superimposed redocked N3 on to the native co-crystallized N3 show a low RMSD of 1.82 Å was observed, whereas redocked VIR250 had a RMSD of 2.096 Å (Supplementary Figures 1b, 2b). These results show less variation in comparison with the native binding pose of ligands in co-crystallized form.

**TABLE 2:** Molecular docking results of food bioactive compounds with COVID-19 main protease (M^pro^), papain-like protease (PL^pro^).

Source	Compounds	M^pro^		PL^pro^	
		Dock score	Polar contacts	Dock score	Polar contacts
Cyanobacteria	Phycocyanobilin	−8.6	Y54, G143, H163, D187, Q189	−9.8	D164 (A), R166 (B), Y264 (A)
Fruits, vegetables, seeds, and grains	Quercetin	−7.8	Y54, Q189	−8	R166 (B), Q269 (A)
Eggs, meat, fruits, and vegetables	Riboflavin	−7.9	E166, C145, H163, L141, S144	−8.5	R166 (A), Y264 (A), Y273 (A)
Grapes and berries	Cyanidin	−7.8	S144, H163	−7.9	E161 (A), Y268 (A)
Legumes	Daidzein	−7.8	T26, E166, Q192, T190	−8.9	K157 (A), D164 (A), R166 (B), Q174 (B)
Legumes	Genistein	−7.6	E166	−8.3	K157 (A), L162 (A), Q174 (B), M208 (B)
Green tea	Catechin	−7.3	L141, H163	−7.1	E161 (B), R166(A)
Grapes and berries skin	Resveratrol	−7	L141, H163, D187	−7.2	R166 (A), E167 (B), C155 (B)
Turmeric	Curcumin	−7	G143, S144, C145	−8	K157 (A), K232 (B), Y264 (A)
Microalgae	Astaxanthin	−7	None	−9.3	G266 (A), M206 (B)
Fruits and vegetables	β-carotene	−6.5	None	−9.2	None
Chili pepper	Capsaicin	−6.3	E166, T190, Q192	−6.5	K157 (A), M208 (B)
Ginger	Gingerol	−6.1	G143, S144, C145, H163, E166	−6.4	R183 (B), E203 (B), R183 (B)
Vanilla	Vanillin	−5	G143, S144, C145, H163, E166	−5.4	Y268 (B), Y273 (B), T301 (B)
Cloves	Eugenol	−4.9	L141, G143, S144, C145, H163	−5.6	S170 (A), C155 (B)
Thyme	Thymol	−4.8	None	−5.4	E203 (B)

To evaluate the reliability of the molecular docking and stability of docked complexes, we conducted molecular dynamics simulations with a traditional force field (CHARMM36m), starting with the docking-generated pose of M^pro^-ligand complexes with binding energy cutoff values of −7.6 Kcal/mol, PL^pro^-ligand complexes with binding energy cut-off values of −8.0 Kcal/mol accompanying with a higher binding affinity toward M^pro^. Root mean square deviation (RMSD) was measured to measure the structural conformation differences between the initial and final positions of proteins and ligands. Table 3 depicts average ligand and protein RMSD and occupancy of major hydrogen bonds for food bioactive compounds in traditional MD simulations. The data show ligand RMSD values are in the range of 0.285 ± 0.090 to 1.743 ± 0.219, protein RMSD values are in the range of 1.370 ± 0.164 to 2.298 ± 0.353 in both M^pro^ and PL^pro^ complexes. These lower RMSD values indicate greater stability of protein-ligand docked complexes.

**TABLE 3:** Average ligand and protein RMSD and occupancy of major hydrogen bonds for food bioactive compounds in traditional MD simulations.

Compound	Ligand-RMSD (Å)	Protein-RMSD (Å)	Major hydrogen bonds and its occupancy (%)
M^pro^			
PCB	1.743 ± 0.219	1.720 ± 0.201	G143 (38.5), N119 (38.7), S46 (25.6), Y54 (12)
Quercetin	0.342 ± 0.104	1.372 ± 0.143	Y54 (18.7), E166 (10.9), S144 (4.5)
Cyanidin	0.832 ± 0.252	1.462 ± 0.207	H163 (34.6), H164 (13.5), G143 (6.7), N (5.9)
Daidzein	0.513 ± 0.145	1.407 ± 0.148	E166 (51.5), R188 (23.9), T190 (25.7), T26 (24.2), G143 (17.4)
Genistein	0.391 ± 0.088	1.370 ± 0.164	D187 (32), E166 (26.8), G143 (9.2), Q189 (4.2)
Riboflavin	1.131 ± 0.193	1.482 ± 0.297	H163 (33.9), N142 (24.7), E166 (15.9), R188 (10.2)
**PL^pro^**			
PCB	1.452 ± 0.125	2.226 ± 0.125	D164(C) (82.1), R166(C) (57.4), D164(A) (51.9), G271(A) (21.6)
Quercetin	0.875 ± 0.118	2.298 ± 0.353	E203(C) (54.5), E167(A) (27.6), S170(C) (22.7), M208(C) (21.4), Y171(A) (8.1), K157(A) (5.5),
Cyanidin	0.285 ± 0.090	1.988 ± 0.316	E203(C) (73.7), Y264(A) (15.1)
Daidzein	0.794 ± 0.088	2.02 ± 0.486	D164(A) (46.6), C155(A) (30.2), K157(A) (19.2), R166(A) (18.1)
Genistein	1.072 ± 0.196	1.864 ± 0.254	Q269(A) (51.9), M208(C) (21), Y171(A) (16.8), K157(A) (11.3)

*Values in parentheses denotes hydrogen bonds occupancy (%).*

### *In vitro* Enzymatic Assay Studies to Screen Potent Phytochemical Inhibitor Compounds Against SARS-CoV-2 M^pro^ and PL^pro^

**FIGURE 2:**
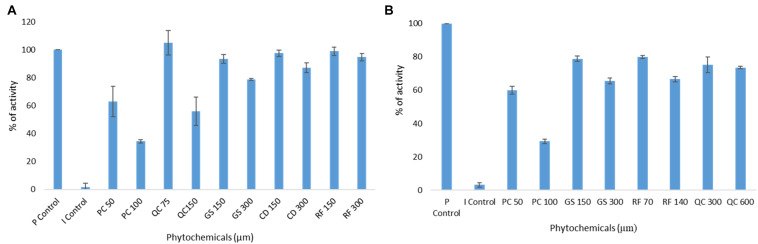
Initial screening of phytochemicals (selected based on docking score and our availability) by *in vitro* enzymatic assays; **(A)** Mpro assay; **(B)** PLpro assay; P control, positive control; I control, inhibitor control; PC, phycocyanobilin; QC, quercetin; GS, genistein; CD, cyaniding; RF, riboflavin.

**FIGURE 3:**
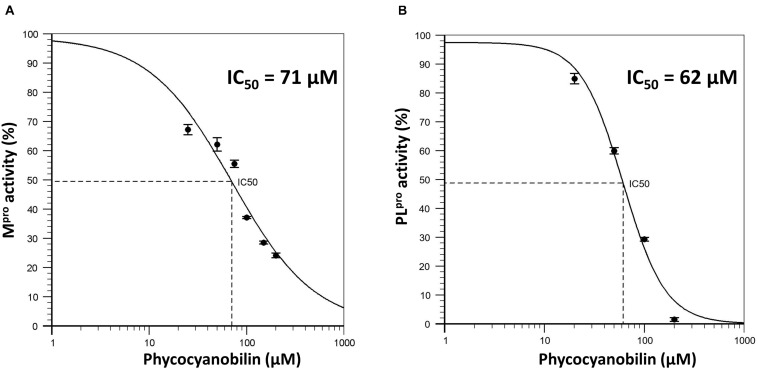
**(A)** Dose response curve of Phycocyanobilin versus M^pro^ activity; **(B)** Dose response curve of Phycocyanobilin versus PL^pro^ activity.

To further validate the molecular docking and molecular dynamics studies, *in vitro* enzymatic studies were conducted. A positive control without the inhibitor compound in the reaction mixture, an inhibitor control that contains authentic inhibitors GC376 (for M^pro^), GRL0617 (for PL^pro^) were used in this study. The enzyme’s relative activity in the presence of inhibitors was estimated by considering positive control activity as 100%. Based on *in silico* studies, we selected the top six phytochemicals (PCB, quercetin, riboflavin, cyanidin, daidzein, and genistein) with a binding energy cutoff value of −7.6 Kcal/mol for M^pro^ enzymatic assay studies. Initial screening results revealed that PCB had higher inhibitor activity followed by quercetin, genistein, cyanidin, and riboflavin (*p* < 0.05) (Figure 2). Further, the IC_50_ value of top two compounds, PCB and quercetin, was determined, and the results show an effective IC_50_ value of 71 μM for PCB (Figure 3) than quercetin (145 μM) (Supplementary Figure 3). For PL^pro^, four compounds (Phycocyanobilin, Riboflavin, Genistein, and Quercetin) with a binding energy cutoff value of −8.0 Kcal/mol, accompanying higher inhibitor activity toward M^pro^, were selected for the *in vitro* inhibitor activity assay (Figure 2). It was envisaged that PCB showed potent inhibitor activity compared to other compounds (Figure 2), with an IC_50_ value of 62 μM (Figure 3). Overall, *in silico* docking and *in vitro* enzyme inhibitor activity data show PCB as a potent inhibitor against SARS-CoV-2 M^pro^ and PL^pro^.

### *In silico* Insights of Broad-Spectrum Inhibitor Activity of PCB Against M^pro^ and PL^pro^

The broad-spectrum efficacy of PCB against CoVs was evaluated by molecular docking studies with available crystal PDB structure of various human and animal CoVs. Table 4 shows the PDB identification code and top docking scores of PCB with M^pro^ and PL^pro^ enzymes of human and animal CoVs. Due to the limitation on the availability of crystal PDB structures of PL^pro^, both dimer and monomeric forms were used in docking studies. For M^pro^, docking scores are in the range of −8.3 to −9.3 Kcal/mol. PCB showed higher binding affinity with docking score (−9.3 Kcal/mol) for MERS M^pro^ followed by HCoV NL63 (−9.0 Kcal/mol) and IBV (−8.9 Kcal/mol). For PL^pro^, docking scores were in the range of −8.9 to −7.6 Kcal/mol. The results reveal that PCB had a higher binding affinity to the dimer form of PL^pro^ enzymes than monomeric forms. When compared monomers only, PCB had best docking score for MERS-CoV (−8.5 Kcal/mol) followed by TGEV (−8.1 Kcal/mol) and SARS-CoV-2 (−8.0 Kcal/mol). Supplementary Figures 4, 5 show polar contacts of PCB with binding pocket key amino acid residues of M^pro^ and PL^pro^ enzymes of human and animal CoVs. Surprisingly, the docking results suggest PCB as a promising broad-spectrum food bioactive inhibitor compound against CoVs proteases.

**TABLE 4:** Molecular docking results of phycocyanobilin with proteases of other pathogenic human and animal CoVs.

CoVs	PDB ID	Dock score	Polar contacts
**M^pro^**			
SARS-CoV-1	1WOF	−8.5	Y54, N142, G143, S144, T190
MERS-CoV	5C3N	−9.3	H41 (2), Q167, K191 (2), Q195 (2)
MHV	6JIJ	−8.4	F138, H161, E164, Q187, Q190
TGEV	2AMP	−8.3	V26, H41, H162
FIPV	5EU8	−8.5	H41, T47, H162, H163, G167, Q191
IBV	2Q6F	−8.9	F46, G141, A142, C143, E187, Q190
HCoV 229 E	3DBP	−8.3	I140, H162, E165, G167
HCoV NL63	5DWY	−9	Y53, G142 (2), A143, H163, Q164
HCoV HKU1	3D23	−8.4	E166 (2), S168
**PL^pro^**			
SARS-CoV-1 (dimer)	2FE8	−8.9	K158 (A), D165 (A), E168 (A), H172 (B)
SARS-CoV-1 (monomer)	2FE8	−7.6	L163, G164, Y269, T302
SARS-CoV-2 (monomer)	6LU7	−8.0	R166, G266
MERS-CoV (monomer)	4RNA	−8.5	D164, D165, G248, G277, Y279
TGEV (monomer)	3MP2	−8.1	D80, H153, Q180, G182, Y184
IBV (monomer)	4 × 2Z	−7.8	D150, F151 (2), S152, D153

### *In silico* Insights Into Inhibitor Activities of Other Phycobilins

**FIGURE 4:**
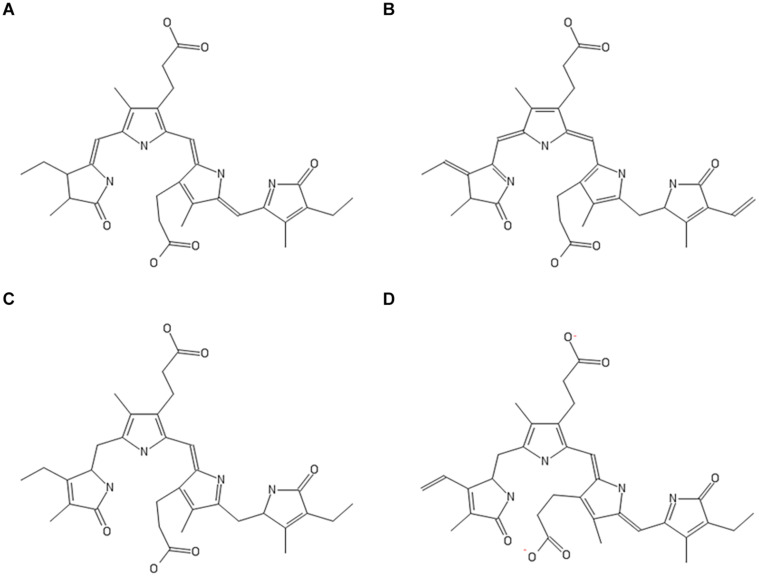
Molecular structures of phycobilins; **(A)** PCB; **(B)** PEB; **(C)** PUB; **(D)** PVB.

Phycobilins are linear tetrapyrrole chromophore compounds found in certain photosynthetic organisms (cyanobacteria, red algae, glaucophytes, and some cryptomonads) and covalently linked to phycobiliproteins (Beale, 1993). Four types of phycobilins are identified: (i) phycoerythrobilin (PEB), (ii) phycourobilin (PUB), (iii) phycoviolobilin (PVB), and (iv) PCB. Figure 4 represents the molecular structures of phycobilins. Based on the PCB results, the other phycobilin inhibitor activity against SARS-CoV-2 proteases via molecular docking approach was demonstrated and docking scores, polar contacts are given in Table 5. All phycobilins show strong binding affinity to key amino acids of M^pro^ and PL^pro^ binding pockets. The docking scores were in the order of PUB (−8.7 Kcal/mol) >PCB (−8.6 Kcal/mol) >PEB (−8.2 Kcal/mol) >PVB (−8.2 Kcal/mol) for M^pro^, whereas in the case of PLpro, the order was PCB (−9.8 Kcal/mol) = PEB (−9.8 Kcal/mol) >PUB (−9.6 Kcal/mol) >PVB (−9.5 Kcal/mol). Nine key binding pocket amino acids (Y54, L141, G143, S144, C145, H163, E166, D187, and Q189) of M^pro^ participated in polar contacts with phycobilins, and specific polar contacts of each phycobilin are shown in Supplementary Figure 6. Ten key binding pocket amino acids [D164 (A), Y264 (A), R166 (A), G266 (A), E161 (A), L162 (A), G271 (A), K232 (A), R166 (B), and T301 (B)] of PL^pro^ participated in polar contacts with phycobilins; specific polar contacts are shown in Supplementary Figure 6.

**TABLE 5:** Molecular docking results of other phycobilins with proteases of SARS-CoV-2.

Other phycobilins	Dock score	Polar contacts
**M^pro^**		
Phycoerythrobilin	−8.2	L141, H163, E166, Q189 (2)
Phycourobilin	−8.7	G143, S144, C145, H163
Phycoviolobilin	−7.7	L141, G143, S144, C145, E166
**PL^pro^**		
Phycoerythrobilin	−9.8	R166 (A), Y264 (A), T301 (B)
Phycourobilin	−9.6	R166 (A) (2), G266 (A)
Phycoviolobilin	−9.5	E161 (A), L162 (A), G271 (A), R166 (B) (2), K232 (A)

## Discussion

Several SARS-CoV-2 mutants are spreading globally; most notably, mutants emerged in the United Kingdom (B.1.1.7), South Africa (B.1.351), and Brazil (P.1) (CDC, 2021). Hence, the efficacy of currently developed vaccines against these variants is questionable. The development of broad-spectrum antiviral drugs against existing viruses is an attractive approach that could provide first-line defense against emerging viral variants. The selection of highly conserved drug targets is an important step to develop broad-spectrum antiviral drugs. Sequence alignment analysis of SARS-CoV-2 and other CoVs (SARS-CoV and MERS) reveals that M^pro^ and PL^pro^ are highly conserved, especially in the functional regions, which makes them potential targets for COVID-19 drug targets (Wu C. et al., 2020).

Several target-based *in silico* screening approaches were performed to find promising protease inhibitors among repurposed drugs, natural phytochemicals, and herbal medicinal compounds against COVID-19 (Amin et al., 2020; Wu C. et al., 2020; Xian et al., 2020). In this study, we focused on screening natural bioactive compounds for potential inhibitor activity against SARS-CoV-2 proteases. Amin et al. (2020) report the list of key amino acids involved in catalysis and substrate binding for M^pro^ (H41, C145, M49, G143, S144, H163, H164, M165, E166, L167, D187, R188, Q189, T190, A191, and Q192) and PL^pro^ (C111, H272, D286, Y268, M208, P247, P248, T301, P248, Y264, N267, Q269, L162, C270, G271, and Y273). Our molecular docking results with natural compounds also show polar interactions in these specific substrate-binding regions for both proteases. Further *in vitro* validation studies found a good correlation between docking and enzymatic assay results. Both *in silico* and *in vitro* approaches show that PCB has strong inhibitor activity against both SARS-CoV-2 proteases. Shih et al. (2003) report direct antiviral activity of allophycocyanin against enterovirus 71 in human rhabdomyosarcoma cells and African green monkey kidney cells. In another study, El-Morsi et al. (2016) demonstrate the reduction of ΦX174 and MS2 by extracted phycobiliproteins from cyanobacterium *Synechococcus cedrorum* Sauvageau.

Though numerous research studies report potential inhibitors with major emphasis on SARS-CoV-2, limited literature is available on broad-spectrum inhibitors against a wide range of CoVs, including human and animal CoVs. For instance, Sheahan et al. (2020) report broad-spectrum antiviral activity of ribonucleoside analog β-D-N4-hydroxycytidine (NHC; EIDD-1931) against SARS-CoV-2, SARS-CoV, MERS-CoV, and bat-CoVs. This study reports the broad-spectrum activity of natural phytochemical compound PCB against 11 CoVs (seven human CoVs and four animal CoVs). The computed physical properties of phycocyanobilin show a rotatable bond count of 10, hydrogen bond donor count of five, and hydrogen bond acceptor count of seven (National Center for Biotechnology Information (NCBI), 2020), which makes multiple hydrogen bond interactions between the compound and specific amino acid residues located at the active site of the pocket of the wide range of protease enzymes. Molecular docking studies indicated that propionic carboxyl and lactam ring carbonyl oxygens of PCB are involved in polar interactions with proteases’ amino acid residues.

To investigate other structurally similar phycobilin (PEB, PUB, and PVB) inhibitor activity, we attempted molecular docking with SARS-CoV-2 proteases. The results reveal that, like PCB, all phycobilins show similar binding affinity toward M^pro^ and PL^pro^ of SARS-CoV-2. Besides this, potent therapeutic properties, such as peroxy radical scavenging, inhibition of cancer cell proliferation, and platelet aggregation are reported for phycobilins (Watanabe et al., 2014). Phycobilin compounds can be directly administered orally as phycobiliproteins (a complex of phycobilins and protein). For instance, when phycocyanin is administered orally to humans, it can be digested and free phycocyanobilin released in the gastrointestinal tract (Watanabe et al., 2014). Thus, noticed therapeutic properties of phycobiliproteins might reflect the effects of their phycobilins (chromophores).

In conclusion, by using *in silico* (molecular docking and MD simulations), *in vitro* enzymatic assay screenings, we discovered PCB as potent phytochemical inhibitors to M^pro^ and PL^pro^ proteases of SARS-CoV-2. Phycocyanobilin had IC_50_ values of 71 and 62 μM for SARS-CoV-2 M^pro^ and PL^pro^, respectively. Further PCB docking studies with other CoVs Mpro and PLpro proteases revealed its broad-spectrum inhibitor activity. A similar binding affinity of other phycobilins (PEB, PUB, and PVB) to these proteases were observed. However, *in vitro* enzymatic studies with M^pro^ and PL^pro^ of other CoVs and *in vivo* studies on the inhibition of CoVs infectivity using human cells and animal models are needed. Further structure-guided development of phycobilin lead compounds could rapidly lead to discovering a single agent with clinical potential against existing and possible future emerging CoV-associated diseases.

## Data Availability Statement

The original contributions presented in the study are included in the article/Supplementary Material, further inquiries can be directed to the corresponding author/s.

## Author Contributions

BP: conceptualization, methodology, investigation, visualization, data curation, and writing original draft preparation. AP: conceptualization, supervision, writing, reviewing, and editing. CD: reviewing and editing. All authors contributed to the article and approved the submitted version.

## Conflict of Interest

The authors declare that the research was conducted in the absence of any commercial or financial relationships that could be construed as a potential conflict of interest.
