# Supplementary material for: Phycobilins as Potent Food Bioactive Broad-Spectrum Inhibitors Against Proteases of SARS-CoV-2 and Other Coronaviruses: A Preliminary Study
Source: Front Microbiol. 2021 Jun 10;12:645713. doi: 10.3389/fmicb.2021.645713 (PMC8222545; doi:10.3389/fmicb.2021.645713)
Supplement: Supplementary file 1 [file Data_Sheet_1.DOCX]

**Phycobilins as potent food bioactive broad-spectrum inhibitors against proteases of SARS-CoV-2 and other coronaviruses: A preliminary study**

**^*^Brahmaiah Pendyala^1^, ^*^Ankit Patras^1^, Chandravanu Dash^2^**

^1^Department of Agricultural and Environmental Sciences, Food Science Program, College of Agriculture, Tennessee State University, Nashville, 37209 TN USA

^2^Meharry Medical College, Nashville, 37208 TN USA

**Supplementary information:**

**
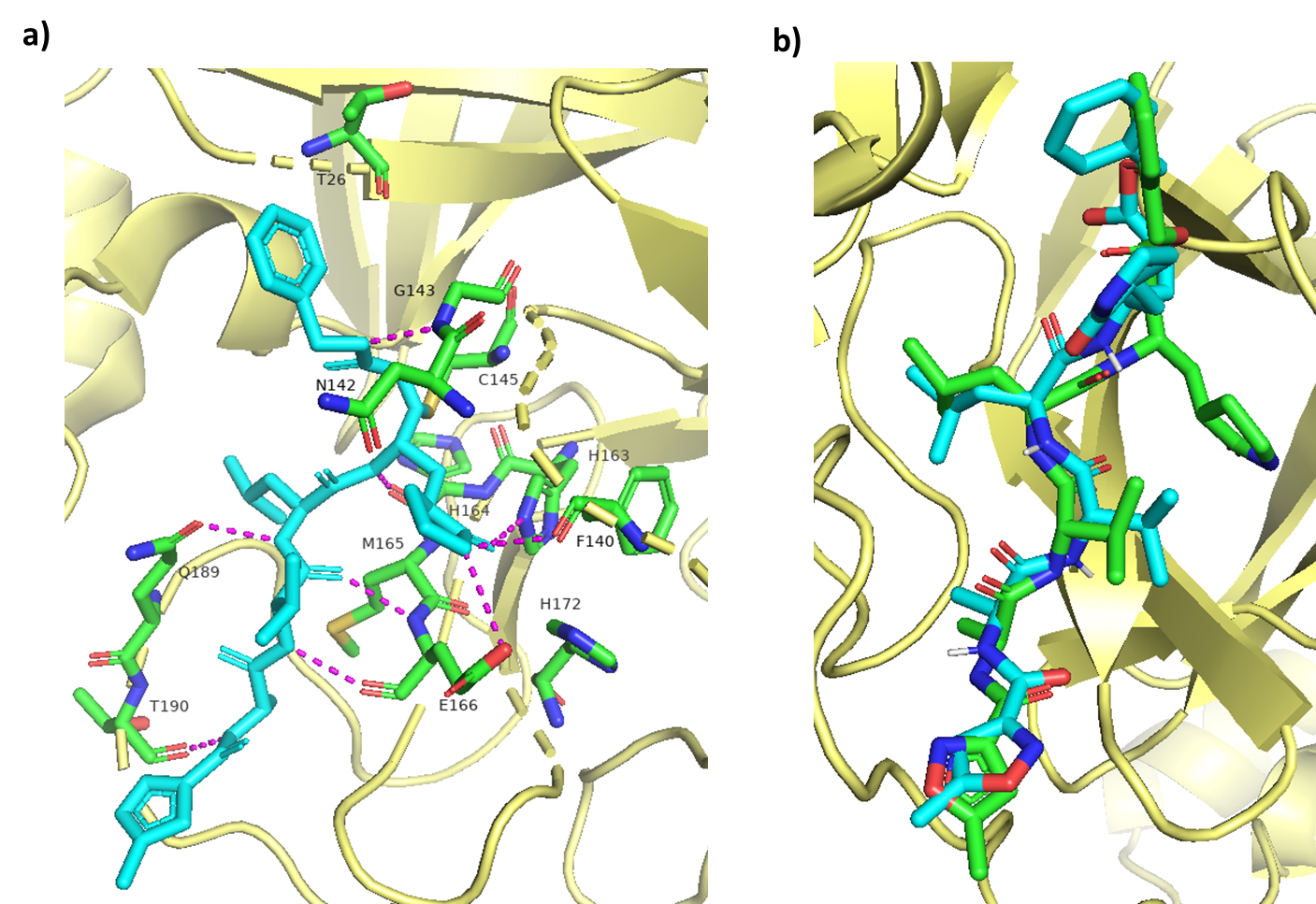
**

**Figure S1: a)** The crystal structure of SARS-CoV-2 M^pro^ in complex with an inhibitor N3. Active site amino acids are represented as green color, polar contacts with protein are indicated in magenta color dotted lines; b) Superpose of redocked N3 with native crystal pose of N3; Cyan color – Native pose, Green color – redocked pose.

**
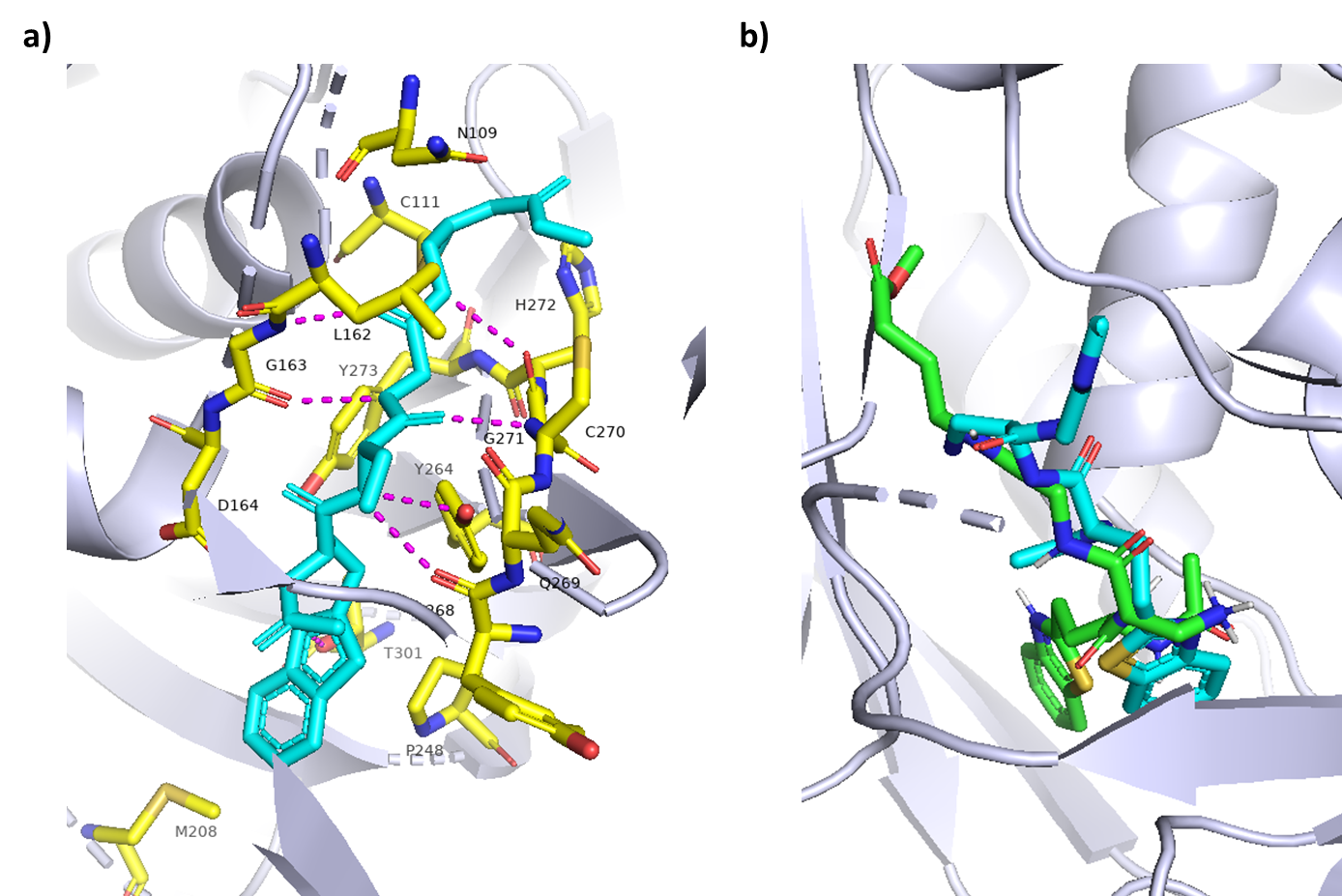
**

**Figure S2: a)** The crystal structure of SARS-CoV-2 PL^pro^ in complex with an inhibitor VIR250. Active site amino acids are represented as yellow color, polar contacts with protein are indicated in magenta color dotted lines; b) Superpose of redocked VIR250 with native crystal pose of VIR250; Cyan color – Native pose, Green color – redocked pose.

.

**
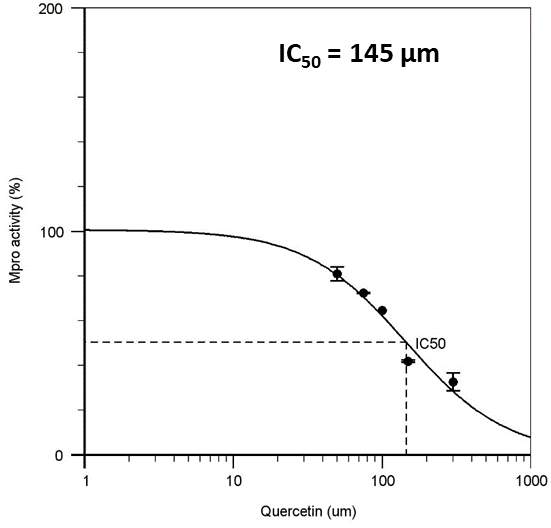
**

**Figure S3**: Dose response curve of Quercetin versus M^pro^ activity


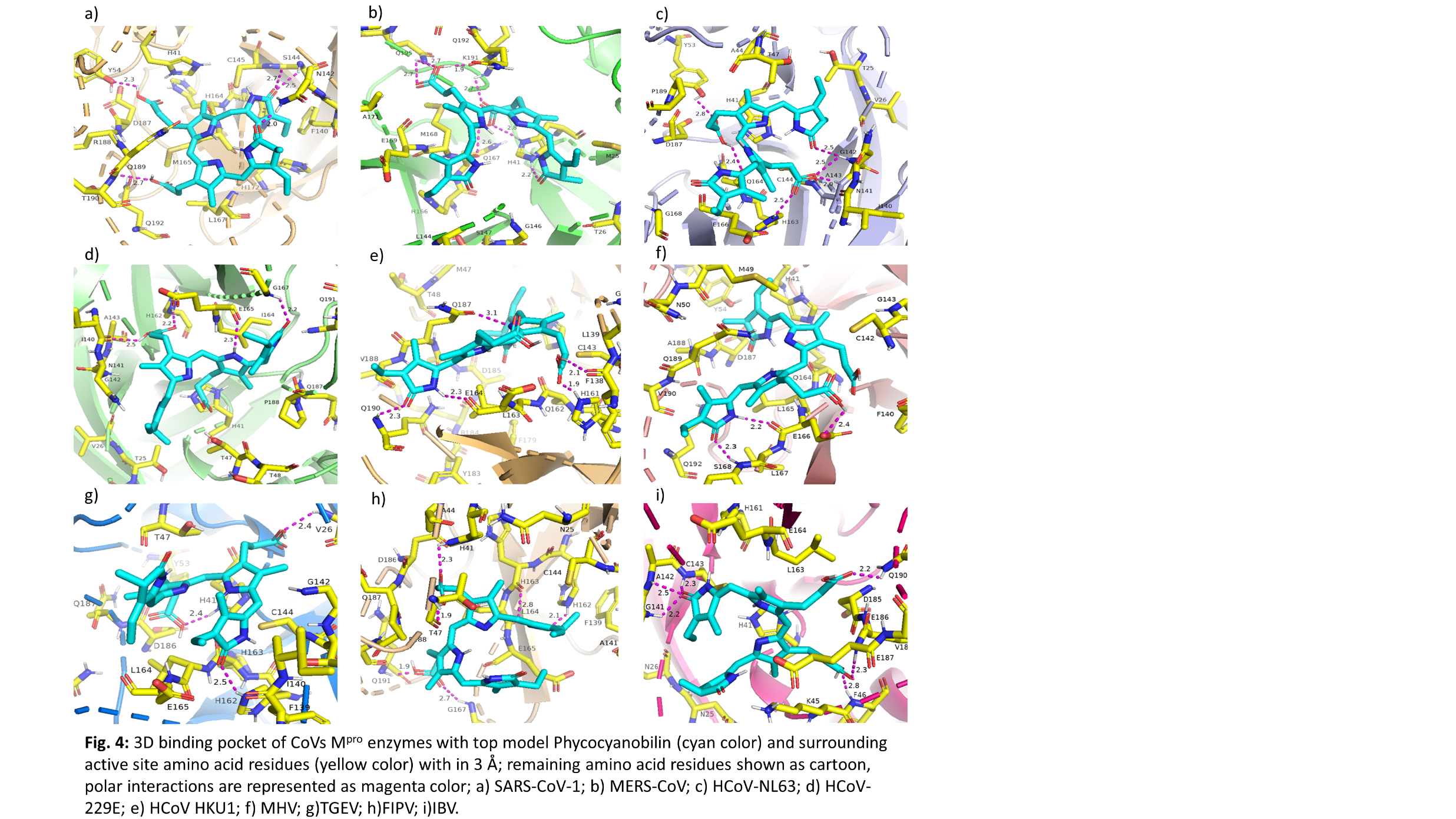


**Figure S4:** 3D binding pocket of CoVs Mpro enzymes with top model Phycocyanobilin (cyan color) and surrounding active site amino acid residues (yellow color) with in 3 Å; remaining amino acid residues shown as cartoon, polar interactions are represented as magenta color; a) SARS-CoV-1; b) MERS-CoV; c) HCoV-NL63; d) HCoV-229E; e) HCoV HKU1; f) MHV; g) TGEV; h) FIPV; i) IBV.


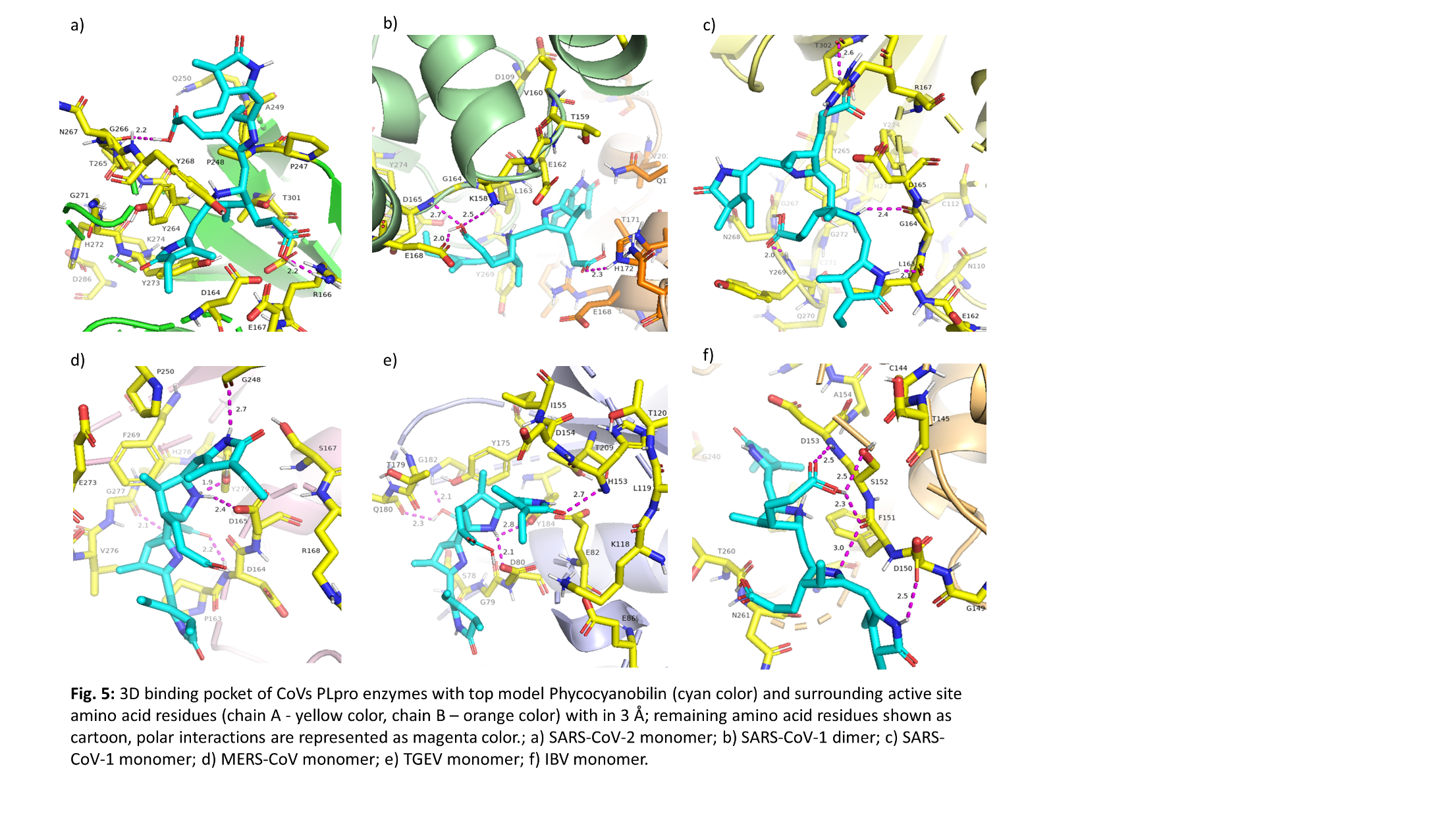


**Figure S5:** 3D binding pocket of CoVs PLpro enzymes with top model Phycocyanobilin (cyan color) and surrounding active site amino acid residues (chain A - yellow color, chain B – orange color) with in 3 Å; remaining amino acid residues shown as cartoon, polar interactions are represented as magenta color.; a) SARS-CoV-2 monomer; b) SARS-CoV-1 dimer; c) SARS-CoV-1 monomer; d) MERS-CoV monomer; e) TGEV monomer; f) IBV monomer.


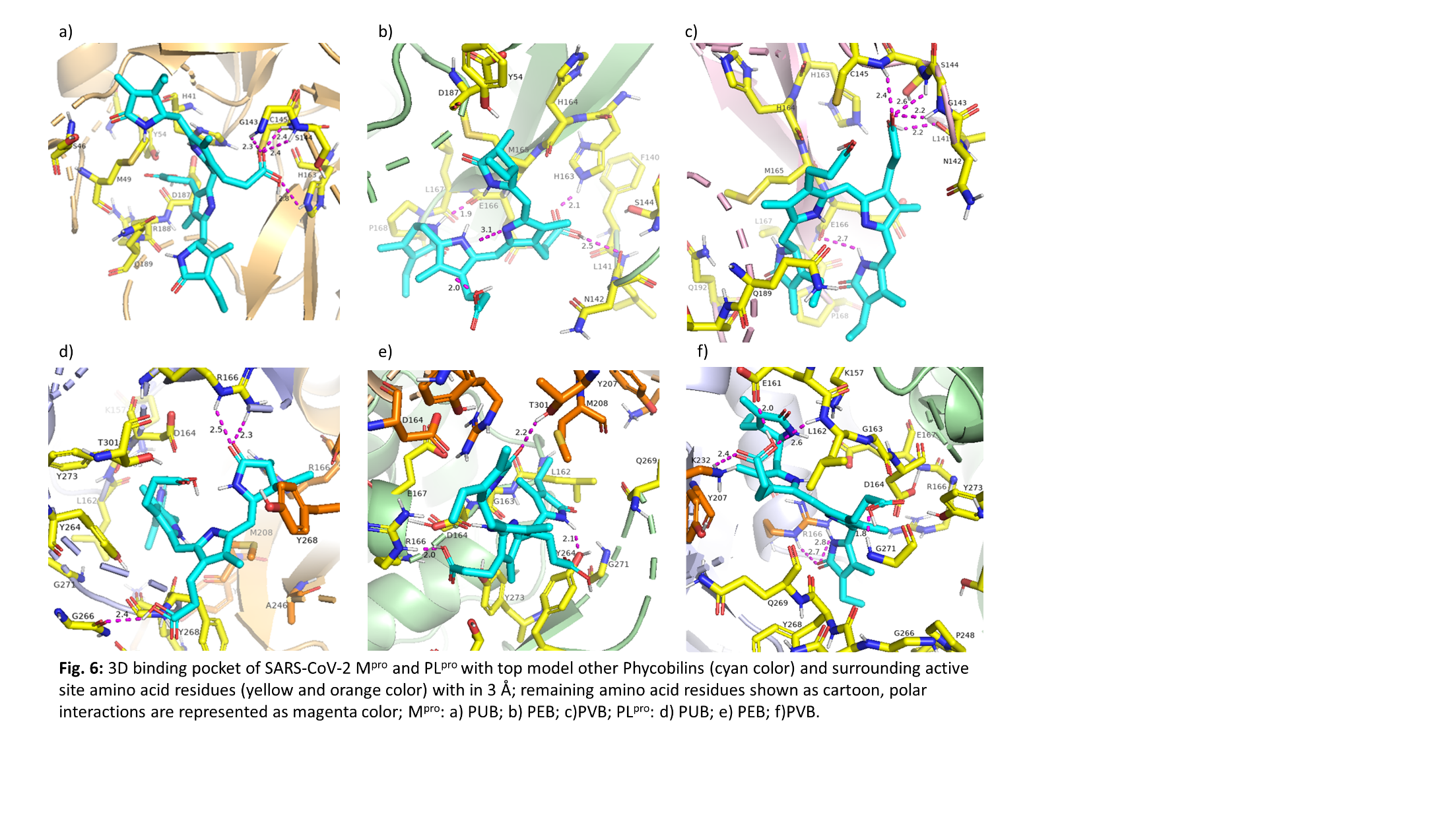


**Figure S6:** 3D binding pocket of SARS-CoV-2 Mpro and PLpro with top model other Phycobilins (cyan color) and surrounding active site amino acid residues (yellow and orange color) with in 3 Å; remaining amino acid residues shown as cartoon, polar interactions are represented as magenta color; Mpro: a) PUB; b) PEB; c)PVB; PLpro: d) PUB; e) PEB; f) PVB.
